# Schrödinger’s Cat Bias: A New Cognitive Bias Proposed

**DOI:** 10.7759/cureus.12697

**Published:** 2021-01-14

**Authors:** José Nunes de Alencar Neto, Eduardo Farina, Maria Catarina Nunes Sampaio

**Affiliations:** 1 Cardiology, Dante Pazzanese Institute of Cardiology, São Paulo, BRA; 2 Scientific Writing Office, Superior School of Sciences of Santa Casa de Misericórdia de Vitória, Vitória, BRA; 3 Cardiology, School of Medicine, University Center Superior Institute of Applied Theology, Sobral, BRA

**Keywords:** cognition, bias, epidemiology

## Abstract

Cognitive biases can cause diverse medical errors and lead to malpractice with potential harm to patients. Some cognitive biases are due to social behavior, professional specialization, and personal experience, leading to commission or omission in medical conduct. We would like to propose a previously undescribed cognitive bias called “Schrödinger’s cat bias.” In 1935, Erwin Schrödinger proposed a dual system based on quantum mechanics that a cat could be dead or alive at the same time. The “Schrödinger’s cat bias” is a situation in which a physician takes a decision and requests an exam or procedure that was unnecessary and puts the patient through an unforeseen risk. After the procedure, if there is a good outcome, the patient will be grateful for it. However, if there is a bad outcome, he would still be grateful for their efforts in trying to find the etiology. This cognitive bias will, most of the time, favor the therapies over the decision of not to treat.

## Introduction

Cognitive biases can be the source of up to 75% of medical errors [1]. We usually see clinicians making a diagnosis or taking clinical decisions based on their clinical expertise. A study demonstrated that 25% of emergency physicians elaborate on their diagnosis hypothesis before meeting the patient and 75% within the initial five minutes of consulting [2].

Kahneman and Tversky proposed a dual-system framework to explain human judgments and decisions [3]. The first path is an easier and quicker way to decide, and it is based on previous experiences. The second path is a more rational and deliberated way, based on critical thinking. Most of the cognitive biases that lead to medical mistakes are because of decisions made relying on the first path, which are clinical guesses that individuals make based on automated thought of previous associations [4]. Another common cause of medical mistakes is due to harmful omissions and commissions. Omissions in medicine are followed with questions such as “Am I right about my diagnosis? Should I wait for a second opinion?” and it can delay emergency situations, just like placing a chest tube in a pneumothorax. On the other hand, commissions usually come along with the following thinking: “better safe than sorry” [5].

With the awareness of the medical community rising on cognitive biases based on over 32 types described [6], we would like to propose a new one called “Schrödinger’s cat bias.”

## Materials and methods

A non-systematic review was conducted searching on the PubMed/MEDLINE database for articles published at any time discussing cognitive biases, particularly in clinical practice. Search terms included “cognitive biases” in the title, abstract, or publication type combined with “clinical practice” in the title or abstract. These terms were further combined with one or more of the following terms in the title or abstract fields: ‘cognitive heuristics’ or “counterfactual thinking”. Resultant articles were cross-referenced for other pertinent articles not identified in the initial search about specific biases, such as commission bias, or were written by eminent scientists in this field. For the examples chosen to illustrate the bias, cases that match the author’s reality and routine as an interventional cardiologist were described and a direct search was performed for the relevant evidence about each case.

## Results

The Schrödinger's cat cognitive bias

In 1935, Erwin Schrödinger proposed an example of quantum mechanics applied in a more complex macroscopic system. The system includes a cat and a radioactive particle which can decay and drop poison that kills the cat. It creates a state of superposition in which the cat is dead and alive at the same time. If the particle has not decayed, the cat is alive; if it did, the cat is dead [7].

The bias proposed in this paper is that an individual, because he does not have the chance to live parallel timelines, does not have the capacity, as he has not got practical experience, to analyze what could have happened to him if he or his doctor had made a different decision. 

This is particularly relevant in medicine. Patients usually overestimate benefits and underestimate risks. If one comes out alive from an intervention, he may feel grateful for that because even if he evolved badly, at least he didn’t live the timeline in which no intervention was performed.

## Discussion

Examples of Schrödinger's cat bias

Situation 1: The Exercise Treadmill Test

The exercise test has a sensitivity of around 60%-70% and specificity around 71% [8], with a positive likelihood ratio of around 2.3 and a negative likelihood ratio of 0.46. 

**Figure 1:**
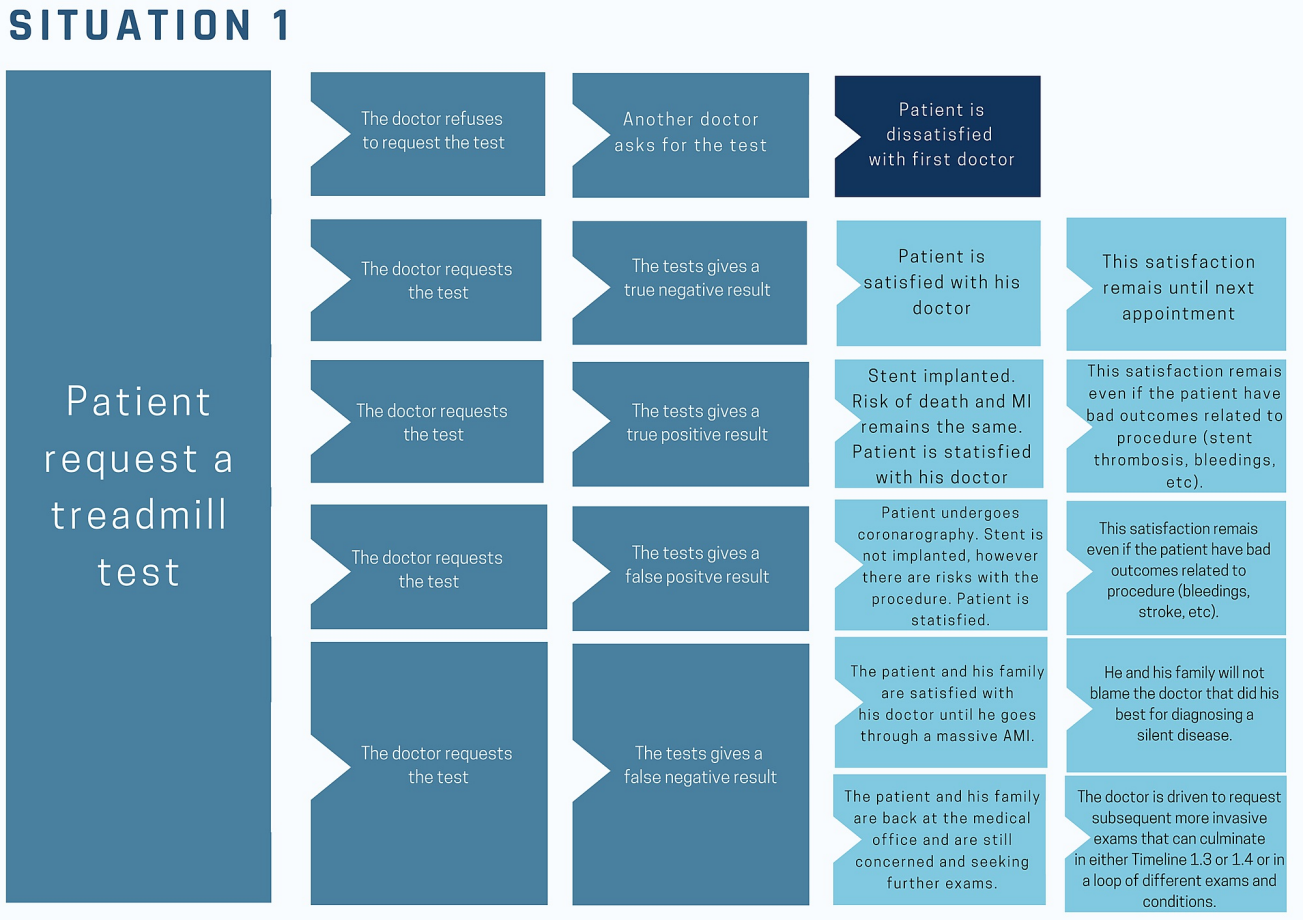
Situation 1 – The exercise treadmill test Different timelines that may happen when a simple treadmill test is asked by the patient. Reader must keep in mind that coronarography, the exam that confirms lesions in coronary artery, is not free from complications (bleeding, stroke, contrast-induced nephropathy) and that a stent may not reduce the risk of having an acute myocardial infarction in future or dying and may have complications (stent thrombosis or restenosis and bleeding).

A patient comes to a medical appointment asking for an exercise treadmill test. There are four possible timelines after this appointment (Figure 1).

Timeline 1.1: The physician refuses to request the exercise test, the patient gets upset, seeks a different doctor who offers the exam, and states: “What could go wrong in an exercise treadmill test? Who knows if there is myocardial ischemia?” The result is negative, and the patient will not go to the original doctor anymore. 

Timeline 1.2: The physician requests the exercise test, and it is negative for ischemia. The patient now feels protected and praises the doctor for taking care of his life.

Timeline 1.3: The physician requests the exam, and there is a true-positive result for ischemia. Now the patient will undergo a percutaneous coronary intervention (PCI). The patient thanks the doctor for finding out about a disease he didn’t feel any symptoms of and could end his life. The patient does not know that PCI probably does not reduce his risk of acute myocardial infarction (AMI) and death [9,10], and only the risk of the procedure remains.

Timeline 1.4: The physician requests the exam, and there is a false-positive result for ischemia. The patient undergoes a coronarography; It does not show any obstructive coronary heart disease, and now the patient feels safe again. 

Timeline 1.5: The physician requests the exam, and there is a false-negative result for ischemia. Two independent timelines may be imagined.

1.5.1: The patient and his family are satisfied with his doctor until he goes through a massive AMI. He and his family will not blame the doctor who did his best for diagnosing a silent disease.

1.5.2: The patient and his family are back at the medical office and are still concerned and seeking further exams. The doctor is driven to request subsequent more invasive exams that can culminate in either Timeline 1.3 or 1.4 or a loop of different exams and conditions.

From the patient’s point of view, it is difficult to know that the physician in Timeline 1.1 was working to avoid unnecessary exams and procedures. Terms like “overdiagnosis” and “overtreatment” are not universally known, and a decision like this may be misinterpreted as omission [11,12]. 

In fact, in all timelines, the risk of an AMI did not change because for AMI to happen, it does not demand previous ischemia, the result of a positive exercise test. Timelines 1.3 and 1.4, however, bring risks for the patient, as coronarography is an invasive exam and there is no clear evidence that invasive treatment [for example, a percutaneous coronary intervention (PCI) is beneficial for decreasing AMI risk or mortality. Timeline 1.3 will presumably be interpreted by the patient as a concerned doctor who saved his life. The subject in Timeline 1.4 will be comforted that the coronarography was negative and possibly will never think that the exam that generated it could never have occurred. Schrödinger’s cat bias almost invariably encourages commission bias [1,13]. This example also illustrates the difficulties that doctors and patients have to recognize and abandon medical reversals, which are previously established standards that were later statistically proved not beneficial: the Schrodinger cat’s bias itself and the fact that contradiction of mainstream practices undermines trust in the medical system [14].

Situation 2: The Surgery

A hypothetical surgery is indicated to a patient because, if not done, the patient will have a five percent risk of dying in 10 years. The patient does not know, but his risk of dying in surgery or the postoperative period is four percent. Over 10 years, one percent of operated patients will die. That means that, at the end of 10 years, there will be no statistical benefit in one strategy over the other.

**Figure 2:**
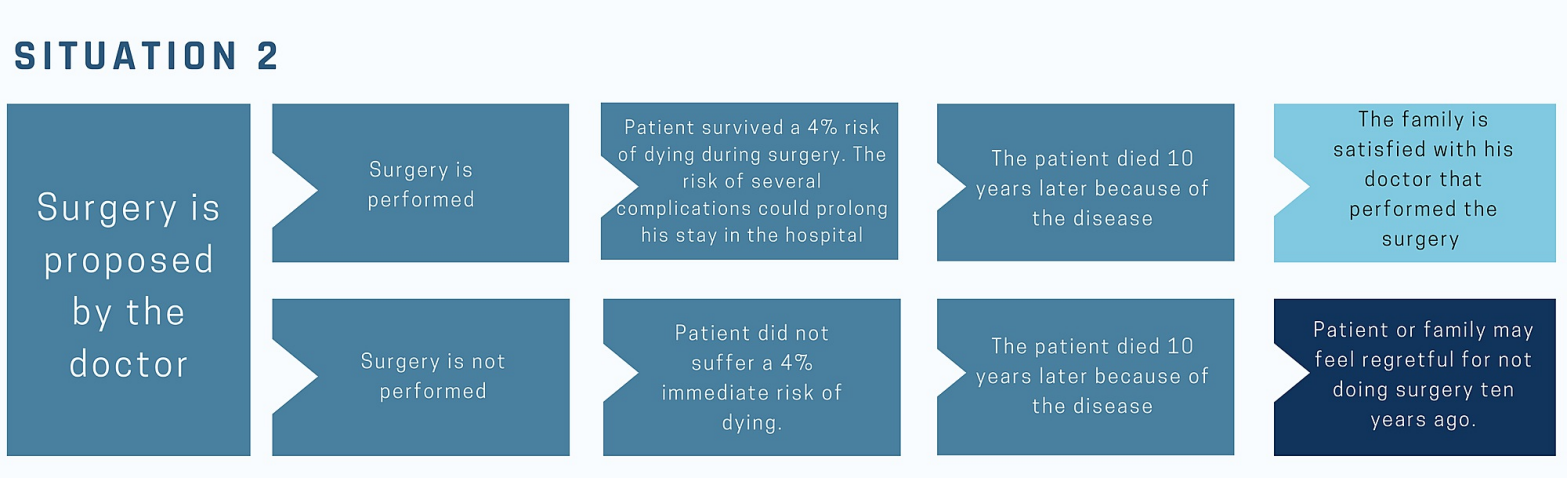
Situation 2 – The surgery Different timelines that may happen when surgery is proposed. This hypothetical surgery depicted poses a 4% risk of dying during surgery. The risk of dying from the disease if surgery is performed is 5% in 10 years, the same risk if the surgery is not performed (1% if survived surgery).

At this time, two distinct timelines can take place (Figure 2).

Timeline 2.1: The surgery is done, and the patient ran an immediate statistical risk of death of four percent but survived. His post-op, however, was problematic. By altered laboratory tests, he started an antibiotic regimen that ended up prolonging his hospital stay. After that, decubitus ulcers appeared. He was discharged 45 days later in a regular condition, requiring home care, which lasted six months. After this period, his statistical risk of death from that condition was one percent over the next 10 years. With six months to go until the 10th anniversary of the surgery, the patient felt sick. The tests revealed a worsening of the disease for which he had been operated. He goes through six hard months, with progressive functional limitations, until he dies.

Timeline 2.2: The surgery is not done, and the patient remained at home, doing his daily activities for nine years and six months. With six months to go before the 10th anniversary of the surgery not done; his illness worsens. He goes through six hard months, with progressive functional limitations, until he dies.

The quality of life described in Timeline 2.2 was superior to that in Timeline 2.1. Besides, the patient in Timeline 2.1 went through a four percent risk of immediate death, which did not happen in Timeline 2.2. A situation like this may be clearly recognizable in surgeries or invasive procedures that lack evidence of statistical benefit. 

Because the patient had the perception that the surgery saved his life - after all the complications, he left the hospital alive - in a few or no moments comes to his mind the possibility of the existence of another timeline in which he had a life free of complications. Note that both timelines had the same five percent accumulated chance of dying. The second timeline, in fact, maybe more correlated with dissatisfaction with the doctor who did not indicate that surgery 10 years ago.

The problem

For many diseases, patients and physicians do not have a probabilistic sense of the problem. For example, the presence of a partial coronary lesion is virtually deterministic in the patient’s perception of an imminent inexorable infarction, although this is not correct. As the patient and the doctor can only live one of these timelines, and especially in the defensive phase of medicine, the tendency is that one thinks more positively in the timeline in which the exam or intervention was performed than in which it was not, even if complications arise.

Counterfactual reasoning is mental representations of alternatives to past events [15]. The bias described in this paper refers to the possibility of the absence of counterfactual reasoning from both patients and physicians, which may drive them to be more susceptible to appreciate testing and therapies regardless of its outcomes and risks, because patients and doctors have excessive and ingenuous reliance on tests and interventions [16]. There are cognitive biases described when counterfactual thinking contributes to unsatisfactory outcomes that may arise when the doctor’s past experiences were traumatic, possibly affecting his decisions in the next comparable case [17]. However, in our literature review, we did not find another reference to a cognitive bias that results from the lack of counterfactual thinking.

## Conclusions

The Schrödinger's cat bias is a new cognitive bias that we propose in this paper that stands for the difficulty that patients and doctors have in imagining their situation if a drug or interventionist therapy had not been indicated. This is due to the fact that, if the patient has gone through treatment, the timeline in which he did not pass through it did not exist, and therefore neither the patient nor the doctor gained practical experience with that other reality. This cognitive bias favors therapies over the decision not to treat, notably at the time of “defensive medicine” in which we live.
